# Research on the Spring Creep Based on the Load Simulator of the Double Torsion Spring Steering Gear

**DOI:** 10.3390/ma16103763

**Published:** 2023-05-16

**Authors:** Bo Zhang, Peijie Ren, Zhuo Wang, Hongwen Ma

**Affiliations:** College of Mechanical and Electrical Engineering, Harbin Engineering University, Harbin 150001, China; zhangbo_heu@hrbeu.edu.cn (B.Z.); wangzhuo_heu@hrbeu.edu.cn (Z.W.); mahongwen@hrbeu.edu.cn (H.M.)

**Keywords:** load simulator, double spring, pre-compression, creep effect, prototype experiment

## Abstract

In this paper, creep at room temperature is studied using a mechanical double−spring steering−gear load table, and the results are used to determine the accuracy of theoretical and simulated data. A creep equation at room temperature, based on the parameters obtained by a new macroscopic tensile experiment method, is used to analyze the creep strain and creep angle of a spring under force. The correctness of the theoretical analysis is verified by a finite−element method. Finally, a creep strain experiment of a torsion spring is carried out. The experimental results are 4.3% lower than the theoretical calculation results, which demonstrates the accuracy of the measurement, with an error of <5% achieved. The results shows that the equation used for the theoretical calculation is highly accurate and can meet the requirements of engineering measurement.

## 1. Introduction

During flight, a missile control system gives instructions to control the wing of the steering gear to rotate at a certain Angle, which changes the direction and magnitude of the gas force acting on the missile, thus changing the missile’s flight trajectory [1]. Therefore, the performance of its steering gear greatly influences the performance of a missile [2]. As steering gear is an integral component of an aircraft system, it is impossible to carry out a steering gear test after the design and manufacture of the system [3]. Therefore, it is necessary to test the performance of steering gear during its manufacture. A simulation load table is mainly used to simulate the force of steering gear during actual use in the environment [4].

At present, large-scale servos are mainly tested using electric and electro-hydraulic servo load simulators [1], while mechanical load simulators are widely used in small servo testing because of their high loading accuracy, small residual torque [5,6], small size, low manufacturing cost, simple structure, and easy maintenance.

For a mechanical load table, a spring torsion bar is generally used as the core component to provide load force. A spring torsion bar has the advantages of small error and high reliability [7]. In order to eliminate the non-linearity of spring reverse loading and the change of stiffness, and to reduce the zero balance range of a spring due to residual stress, friction, zero hysteresis, and other factors, Zhang [8] has proposed a mechanical load table with a structure of a double-spring coaxial reverse arrangement and pre-compression, which can effectively reduce the zero balance range. Because of the moment produced by the pre-compression of a spring, the spring will creep.

Zhu [9] studied the creep process of a precision helical tensile spring, which provided a method for measuring the creep of a tensile spring at room temperature. T. H. Alden [10] studied the strain hardening of 304 stainless steel during low temperature creep and proposed a theory that can be used to predict the creep curve and the hardening effect caused by creep. A. Oehlert [11] studied the room-temperature creep of high strength steel and found that creep can occur at lower stresses and that creep strain increases with creep time and stress, but decreases with an increase of number of cycles. B. Alfredsson [12] conducted low-temperature creep experiments on martensite and bainite microstructures of high-strength rolling bearing steel, and found that the two exhibited different primary creep behaviors. The research results have certain guiding significance for the design and application of high-strength steel. Paul R. Barrett [13] developed a modified Coble creep model to describe the experimental low-stress creep rates in alloys with thermally stable precipitate structures. Brian K. Milligan [14] has studied the creep behavior of Al-Cu alloys at certain temperatures, and found that increasing the thermal stability of the precipitates in Al-Cu alloys can significantly improve their creep properties. Hu [15] explained how the evolution of microstructure affects the creep properties of a material physically, and evaluated several secondary phenomena in the curve of creep rate versus time of 316H stainless steel, which is vital to the realistic life assessment of critical engineering components. Wu [16] established the creep constitutive equation of a stainless steel spring to study the creep of a spring, and found that the higher the ambient temperature, the greater the creep strain of a stainless steel spring. When the service temperature increases from 25 °C to 320 °C, the 24h creep strain increases by five times.

At present, the research on creep mainly focuses on the creep behavior of materials, or the establishment of creep models under high temperature, but the research on creep at room temperature is relatively scarce. Most of the research on the creep behavior of springs has been carried out at high temperature and mainly focus on the stress relaxation of a spring [17,18,19,20,21,22]. In this paper, the influence of creep effect of a spring due to pre-compression is studied. The specific research contents are as follows: Through deconstruction and reorganization, using the original room-temperature creep constitutive equation as a basis, a room-temperature creep constitutive equation of a torsion spring with relevant parameters is obtained based on a macro-tensile test, which is then compared and verified by a finite-element simulation. The stress and strain of a spring are analyzed, and an accurate stress expression of the spring is obtained. Experiments are designed to verify the creep performance of a spring, and the error between the theoretical calculation results of spring creep strain and experimental data is obtained.

## 2. Creep Equation and Experimental Method of a Spring at Room Temperature

### 2.1. Creep Equation of a Spring at Room Temperature

In room-temperature creep, the creep deformation increases logarithmically with time, which is consistent with the first stage of typical creep. Strain hardening and fatigue models are usually used in creep theory at room temperature. The fatigue model is more accurate in fast loading, while the strain hardening model can be used in room-temperature creep under arbitrary loading [23].

According to the microscopic situation of room-temperature creep, Schoeck [24] proposed the constitutive equation of room-temperature creep:

(1) $${\dot{\varepsilon }}_{c}=NAV\nu \exp(-\frac{U}{KT}),$$

where: $\dot{\varepsilon_{c}}$—room-temperature creep rate; N—dislocation density; *A*—the area of dislocations swept after passing an obstacle; *V*—activation volume; ν—vibration frequency of the dislocation line; U—thermal activation energy required to pass obstacles; K—Boltzmann constant; *T*—experimental temperature.

U can be expressed as the product of the thermal activation energy, $U_{0}$, minus the effective stress, $\sigma_{eff}$, acting on the dislocation line and the activation volume, V, namely:

(2) $$U=U_{0}-\sigma_{\mathrm{eff}}V.$$

In the strain hardening model, the external stress, σ, is constant, but due to the hardening effect, the effective stress, $\sigma_{eff}$, decreases with the increase of creep value, $\varepsilon_{c}$, namely:

(3) $$\sigma_{\mathrm{eff}}=\sigma -\theta \varepsilon_{c},$$

where: θ—hardening coefficient at room-temperature creep; $\varepsilon_{c}$—creep value.

The relationship between room-temperature creep and creep time can be obtained by introducing Equations (2) and (3) into Equation (1), namely:

(4) $$\varepsilon_{c}=\alpha \ln(\frac{t_{c}}{\tau }+1),$$

where:

(5) $$\alpha =\frac{KT}{\theta V},\tau =\frac{KT}{\theta V}\frac{1}{NAB\nu }\exp\frac{U_{0}-\sigma V}{KT},$$

where: *B*—Burgers vector; θ—strain hardening coefficient.

Derived from Equation (4), the relationship between creep rate, $\dot{\varepsilon_{c}}$, and creep time, $t_{c}$, at room temperature can be obtained as follows:

(6) $${\dot{\varepsilon }}_{c}=\frac{\alpha }{t_{c}+\tau }.$$

After the end of loading, creep just appears. At this time, $t_{c}=0$ and $\dot{\varepsilon_{c}}=\alpha /\tau =\dot{\varepsilon_{c0}}$, which can be substituted into Equation (6) to obtain the following relationship:

(7) $${\dot{\varepsilon }}_{c}={\left(\frac{t_{c}}{\alpha }+\frac{1}{{\dot{\varepsilon }}_{c0}}\right)}^{-1}.$$

It can be seen from Equation (7) that the main influencing factors of creep rate are α and $\dot{\varepsilon_{c0}}$, which can be directly obtained by experiment. By fitting the experimental data with Equation (7), the creep rate equation can be obtained.

According to Equation (6), the room-temperature creep rate, $\dot{\varepsilon_{c}}$, can be obtained only after obtaining the influencing factors α and τ. However, these two factors are a measure of micro performance, which are difficult to obtain and not suitable for the situation of large individual differences. Therefore, Xiao [24] adopted a method to calculate room-temperature creep only with macro parameters, and the relevant parameters can be obtained through routine experiments, which is a more simple and convenient method in engineering applications.

The initial creep rate, $\dot{\varepsilon_{c}}=\alpha /\tau =\dot{\varepsilon_{c0}}$, at the beginning of creep can be combined with Equation (4) to obtain Equation (8):

(8) $$\varepsilon_{c}=\alpha \ln(\frac{t}{\alpha }{\dot{\varepsilon }}_{c0}+1).$$

Therefore, the parameter *τ* is transformed into the initial creep rate, $\dot{\varepsilon_{c0}}$. There is no difference between creep loading at room temperature and tensile-test loading. Therefore, the strain rate at the moment when the room-temperature creep loading is completed is equal to the rate when the creep is just carried out, and the creep stress is equal to the stress at the end of the loading.

The Ramberg–Osgood model [25] is usually used to describe the stress–strain curve of steel. This model was put forward in 1943. The main idea is that the strain of a material is composed of elastic deformation and plastic deformation. The nominal flow limit, $\sigma_{0.2}$, of a material is selected by the classical method, and the corresponding deformation $\varepsilon_{0.2}$ = 0.002, then the equation for the Ramberg–Osgood model is:

(9) $$\varepsilon =\frac{\sigma }{E}+0.002{\left(\frac{\sigma }{\sigma_{0.2}}\right)}^{n},$$

where: $\sigma_{0.2}$—nominal flow limit; *n*—strain hardening coefficient.

The strain hardening coefficient can be selected by the classical method. If $\sigma {=\sigma }_{0.1}$ is used [26], the strain hardening coefficient is:

(10) $$n=\frac{\ln(\varepsilon_{0.2}/\varepsilon_{0.1})}{\ln(\sigma_{0.2}/\sigma_{0.1})}$$

The above equation is accurate when the stress is less than the nominal flow limit, $\sigma_{0.2}$, but when the stress exceeds the nominal flow limit the calculated result of this model is larger than the actual result.

On the basis of the Ramberg–Osgood model [25], Kim J. R. Rasmussen [27] put forward the method of subsection fitting through experimental research. The boundary point is the nominal flow limit, $\sigma_{0.2}$. When the stress is less than the nominal flow limit, the Ramberg–Osgood model is used. After $\sigma_{0.2}$ is exceeded, the Ramberg–Osgood model is calculated in the translation coordinate system. Through a large number of experimental calculations and statistical analysis, an improved Ramberg–Osgood model is obtained:

(11) $$\varepsilon =\left\{ \begin{array}{l} \frac{\sigma }{E}+0.002(\frac{\sigma }{\sigma_{0.2}}{)}^{n},\sigma \leq \sigma_{0.2}\\ \frac{\sigma -\sigma_{0.2}}{E_{0.2}}+\varepsilon_{u}(\frac{\sigma -\sigma_{0.2}}{\sigma_{u}-\sigma_{0.2}}{)}^{m}+\varepsilon_{0.2},\sigma \geq \sigma_{0.2} \end{array}.\right.$$

(1) When $\sigma \leq \sigma_{0.2}$, n is the strain hardening coefficient, which can be calculated by Equation (10).
(2) When $\sigma \geq \sigma_{0.2}$, $E_{0.2}$ is the initial Young’s modulus at this stage, that is, the tangent modulus at 0.2% yield strength. Its value can be calculated by Equation (12):

(12) $$E_{0.2}=\frac{E}{1+0.002n/e},$$

where: *e*—parameter, $e=\sigma_{0.2}/E$; $\varepsilon_{u}$—total strain at final fracture; $\sigma_{u}$—stress at final fracture, i.e., tensile strength; *m*—index, m = 1 + 3.5$\sigma_{0.2}/\sigma_{u}$; $\varepsilon_{0.2}$—$\sigma_{0.2}$ corresponding total engineering strain, $\varepsilon_{0.2}=\sigma_{0.2}/E+0.002$.

The strain rate at the loading stage can be obtained by deriving the time *t* from both sides of Equation (11) at the same time.

(13) $$\frac{d\varepsilon }{dt}=\left\{ \begin{array}{l} {}[\frac{1}{E}+0.002n(\frac{\sigma }{\sigma_{0.2}}{)}^{n-1}]\frac{d\sigma }{dt},\sigma \leq \sigma_{0.2}\\ {}[\frac{1}{E_{0.2}}+\varepsilon_{u}m(\frac{\sigma -\sigma_{0.2}}{\sigma_{u}-\sigma_{0.2}}{)}^{m-1}\frac{1}{\sigma -\sigma_{0.2}}]\frac{d\sigma }{dt},\sigma \geq \sigma_{0.2} \end{array}\right..$$

It is known that the state at the end of loading is the initial state at the beginning of creep, that is, $\dot{\varepsilon_{c0-}}=\dot{\varepsilon_{c0+}}=\dot{\varepsilon (T_{1})}$, and $\dot{\varepsilon_{c0}}=\alpha /\tau$. By substituting Equation (8), Equation (14) can be obtained:

(14) $$\varepsilon_{c}=\alpha (\sigma_{c})\ln[1+\frac{t}{\alpha (\sigma_{c})}{\dot{\varepsilon }}_{1}(\sigma_{c})],$$

where $\sigma_{c}$ is the constant stress in the creep stage of the material and its value is equal to the material stress at the completion of loading. Therefore, $\dot{\varepsilon (\sigma_{c})}=\dot{\varepsilon (T_{1})}$. By using Equations (12) and (13), the creep value increases with the increase of creep time.

### 2.2. Spring Material and Size Parameters

The spring material is 65 Mn, and its specific performance parameters are shown in Table 1.

The spring calculation process has been mentioned in another article [8], and the spring size parameters are shown in Table 2 below.

### 2.3. Calculation of Temperature Creep in Spring Chamber

According to article [28], the maximum stress of a cylindrical helical torsional spring when it only withstands external torque T is:

(15) $$\sigma_{\mathrm{bb}}=-\frac{\cos^{3}a}{z_{m}C}T[0.154+(0.246\cos^{2}a-0.096\sin^{2}a)\frac{1}{C}],$$

(16) $$\sigma_{\mathrm{tt}}=-\frac{\cos\alpha }{z_{m}}T[1+0.871\frac{\cos^{2}\alpha }{C}+(0.032\sin^{2}\alpha +0.642\cos^{2}\alpha )\frac{\cos^{2}\alpha }{C}],$$

and

(17) $$\tau_{\mathrm{tb}}=\tau_{\mathrm{bt}}=\frac{\sin\alpha }{z_{t}}T[1+0.635\frac{\cos^{2}\alpha }{C}+0.163\frac{\cos^{4}\alpha }{C^{2}}],$$

where: a—torsion spring mounting Angle; $z_{m}$—flexural section coefficient; C—spring index; $z_{t}$—torsion section coefficient; α—helical angle.

According to Mohr’s strength theory, the equivalent stress at the danger point of the spring is:

(18) $$\sigma^{*}=\frac{1-m}{2}(\sigma_{\mathrm{tt}}+\sigma_{\mathrm{bb}})+\frac{1+m}{2}\sqrt{{\left(\sigma_{\mathrm{tt}}-\sigma_{\mathrm{bb}}\right)}^{2}+4\tau_{t}^{2}}$$

and

(19) $$m=\frac{\sigma_{\mathrm{st}}}{\sigma_{\mathrm{sc}}}\leq 1$$

where: $\sigma_{st}$—tensile yield point; $\sigma_{sc}$—compressive yield point.

When the spring is compressed by 30°, the moment of a single spring is 300 N·mm. By substituting the relevant values in Table 2 into Equations (15)–(17), $\sigma_{bb}$ = 16.869 MPa, $\sigma_{tt}$ = 539.33 MPa, and $\tau_{bt}$ = 20.99 MPa can be obtained. By substituting these three values into Equation (18) (where m = 0.9231), the equivalent stress, $\sigma^{*}$ = 525.38 MPa, of the spring danger point can be obtained.

Since the equivalent stress $\sigma^{*}$ = 525.38 MPa is $\leq \sigma_{0.2}$ at the spring danger point, the strain rate at the spring danger point under constant external load can be obtained by substituting the parameter into Equation (13).

(20) $$\frac{d\varepsilon }{dt}=[\frac{1}{E_{0}}+\frac{0.002n}{\sigma_{0.2}}{\left(\frac{\sigma }{\sigma_{0.2}}\right)}^{n-1}]\frac{d\sigma }{dt}$$

**Figure 1:**
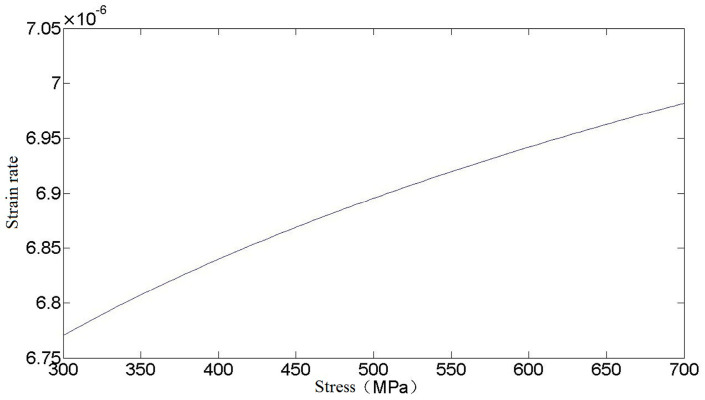
Relationship between stress and strain rate.

When the equivalent stress $\sigma \leq \sigma_{0.2}$, in the loading stage, the relationship between stress and strain rate is given by Equation (20). Assuming that the loading rate is 1, the relationship between them is shown in Figure 1.

Upon substitution of the equivalent stress of the spring danger point into Equation (20), and applying the result to Equation (14), the calculation formula for creep strain under external load T = 300 N∙mm is:

(21) $$\varepsilon_{c}=\alpha (\sigma_{c})\ln[1+\frac{t}{\alpha (\sigma_{c})}(\frac{1}{E_{0}}+\frac{0.002n}{\sigma_{0.2}}{\left(\frac{\sigma }{\sigma_{0.2}}\right)}^{n-1})\frac{d\sigma }{dt}].$$

In Equation (21), n and $\alpha (\sigma_{c})$ can be obtained by fitting the data obtained from tensile and creep tests, where *n* = 1.113 and $\alpha (\sigma_{c})$ is:

(22) $$\alpha (\sigma_{c})=8.1427\times 10^{-9}\sigma_{c}.$$

According to the relevant experiment experience of loading rate, in the tensile test, the value of loading rate is generally set at 5~40 MPa/min. According to relevant literature [29], the magnitude of strain in the first stage of the material obtained from loading rates within the range of 5~40 MPa/min remains basically unchanged, with slight differences in subsequent stages, but the difference is not significant. Therefore, to simplify the calculation, the loading rate is selected as 20 MPa/min, that is, dσ/dt= 0.333 MPa/s.

By integrating Equations (14), (20) and (22), and taking the loading rate as 0.333 MPa/s, the expression formula of creep stress can be obtained as follows:

(23) $$\varepsilon_{c}=\alpha (\sigma_{c})\ln[1+\frac{t}{\alpha (\sigma_{c})}(\frac{1}{3E}+\frac{0.002n}{3\sigma_{0.2}}{\left(\frac{\sigma_{c}}{\sigma_{0.2}}\right)}^{n-1})].$$

**Figure 2:**
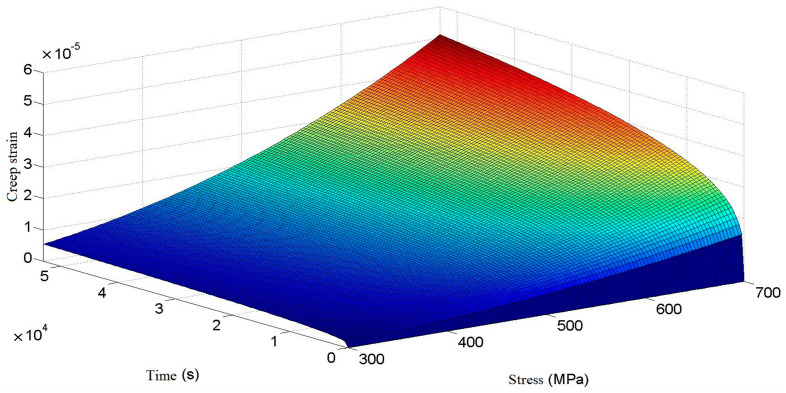
Relation of creep strain to stress and time.

It can be seen from the above formula that creep strain is mainly related to time and stress, and the relationship between the three is shown in Figure 2.

As can be seen in Figure 2, the room-temperature creep of a cylindrical helical torsion spring shows a typical creep curve trend when the stress is determined. In the case of low stress, the creep of the torsion spring enters the second stage of stable creep in a short time. In the stable creep stage, the creep strain rate is small, and there is little increase in creep strain with time. In the condition of high stress, the first stage of creep of a torsion spring ends after a longer time, and the creep of the torsion spring enters the second stage of stable creep after a longer time. It can be seen that, in the same case, the greater the stress produced by a torsion spring, the longer the time it experiences in the first creep stage. In the stable creep stage, compared with the lower stress condition, the creep strain rate is larger, and the increase of creep strain is larger for a long time.

**Figure 3:**
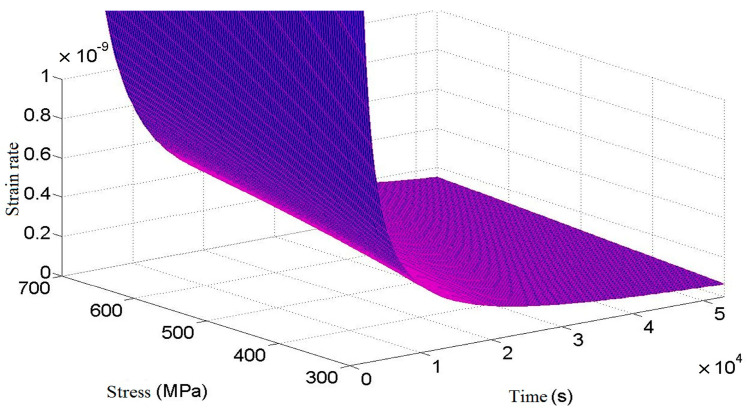
Relation of creep rate to stress and time.

After derivation of time t on both sides of Equation (23), the relationship between creep rate of a cylindrical helical torsional spring, stress, and time can be obtained, as shown in Figure 3. As can be seen from Figure 3, the creep rate decreases significantly with the increase of time. Compared with a state of low stress, the time of torsional spring creep in the first stage of creep increases obviously in a state of high stress, and the creep rate in the second stage of creep also increases obviously.

**Figure 4:**
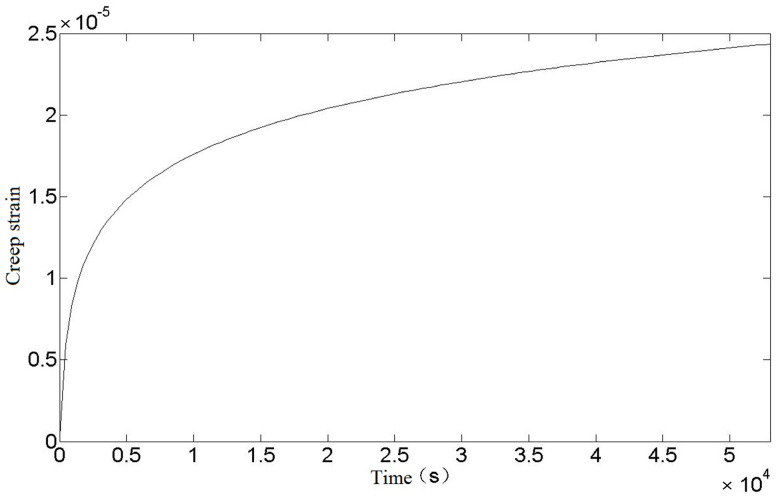
Relationship between creep strain and time of torsion spring.

Since the stress values in each part of the spring are not equal and cannot be calculated in detail, it is not practical to calculate the creep strain. In order to simplify the calculation and improve the safety margin, the creep strain values at the spring danger points are chosen to replace the creep strain values at each part of the spring. It can be seen from the above that the equivalent stress of the spring danger point is $\sigma^{*}$ = 525.38 MPa, and the relationship between creep strain value and time can be obtained by substituting it into Equation (23), as shown in Figure 4.

It can be seen from Figure 4 that the creep strain curve after the torsional spring loading is in line with the first and second stages of the theoretical creep curve. With the increase of time, the creep strain continues to increase and the rate decreases to a fixed value. The creep strain of the torsional spring will not enter the third stage because it is at room temperature and the loading stress is not large.

In the elastic deformation stage, according to data [30], the stress–strain relationship in pure bending can be written as:

(24) $$\varepsilon_{e}=\frac{(\rho +y)d\theta -\rho d\theta }{\rho d\theta }=\frac{y}{\rho },$$

where: *y* is the distance between the linear strain on the section and the neutral axis, and assuming that each fiber is only subject to axial tension and compression, it can be obtained according to Hooke’s law:

(25) $$\sigma =E\varepsilon =E\frac{y}{\rho }$$

and

(26) $$\frac{1}{\rho }=\frac{M}{EI_{z}}.$$

Therefore, the stress–strain relationship at the lower boundary of the section in the elastic stage of a torsion spring is:

(27) $$\varepsilon_{e}=\frac{yM}{EI_{z}}.$$

**Figure 5:**
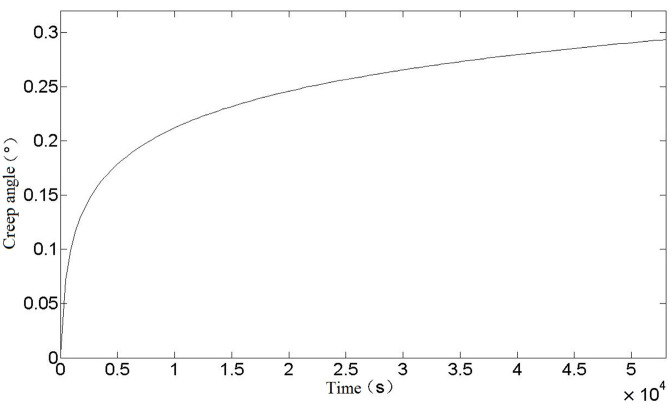
Relationship between creep angle and time of torsion spring.

According to the relationship between creep strain and elastic strain of a torsion spring, the change of rotation angle during creep of a torsion spring can be obtained, as shown in Figure 5.

In this paper, the creep process of a torsion spring is calculated theoretically, and the creep strain of a torsion spring at room temperature, and the relationship between creep angle and time, are obtained. The following uses the finite-element method to simulate the creep process of a torsion spring.

**Figure 6:**
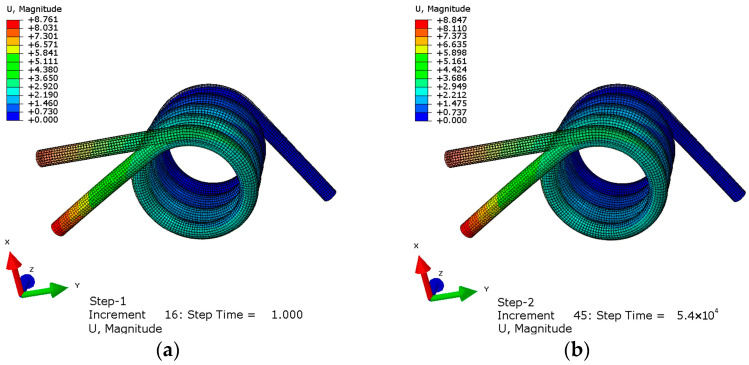
Torsion spring deformation diagram: (**a**) elastic deformation diagram (**b**) creep deformation diagram.

The simulation was carried out by using Abaqus. One end of the torsion spring was fixed, and a torque of 300 N∙mm was applied to the other end. In the first analysis step, a torque of 300 N∙mm was applied to make the spring undergo elastic deformation, and the time was 1 s. The second analysis step was creep analysis, which lasted for 54,000 s. The displacement results obtained are shown in Figure 6.

**Figure 7:**
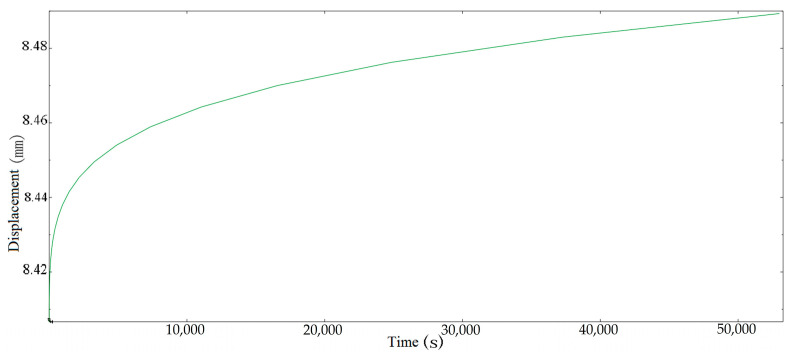
Creep simulation displacement of torsion spring.

The shadow in Figure 6 is the image before the torsion spring is deformed. It can be seen from the figure that the displacement at the elastic deformation stage is 8.761 mm from the farthest point of the central axis of the spring. After 54,000 s, its deformation increases to 8.847 mm. Compared with the elastic stage, the creep deformation is 0.98% of the elastic deformation and the creep strain is ${2.45\times 10}^{-5}$. In order to display the creep curve more clearly, the curve at the elastic deformation stage is ignored and only the curve within a period of time at the beginning of creep is truncated. The point is selected as the lower endpoint of the torsional spring applying force, and the creep curve is shown in Figure 7.

**Figure 8:**
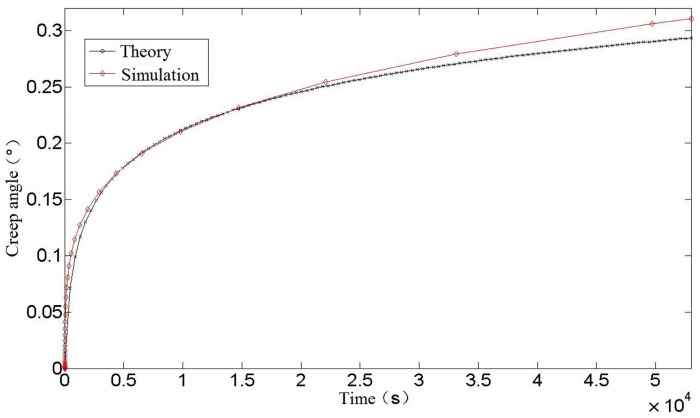
Comparison between theory and Simulation of torsion spring.

It can be seen from Figure 7 that the creep simulation curve of a torsion spring is similar to the theoretical calculation curve, and a comparison between the simulation curve and the theoretical curve is shown in Figure 8.

It can be seen from Figure 8 that the theoretical calculation value of torsion spring creep is slightly less than the simulation value in the early stage, and the theoretical calculation value is slightly greater than the simulation result as time goes on. In the later period, the theoretical calculation is smaller than the simulation result. At 54,000 s, the theoretical calculation value of torsion spring creep is 0.2935°, while the simulation result is 0.3105°, which is 5.79% larger than the theoretical calculation, proving that the theoretical calculation formula is more accurate. Regarding the error between the theoretical calculation and simulation results: on the one hand, it may be because the software adopts traditional age-hardening creep theory in the finite-element simulation process, without considering the influence of some material properties, such as interaction and microstructure. In addition, when using this theory, parameters such as creep strain rate, creep activation energy, and initial hardness of materials need to be determined. If the actual material parameters are different from those used in the theoretical calculation, the calculation results will be biased. On the other hand, it may be because some small quantities are omitted in the derivation of theoretical formulas, which leads to the change of calculation accuracy. However, on the whole, the error between the theoretical calculation results and the finite-element simulation results is within an acceptable error range, which shows that the theoretical calculation results are more accurate and can accurately predict and estimate the performance and life of a spring in use, which is of great significance for designing high-performance and reliable spring components.

### 2.4. Experimental Method of Spring Chamber Temperature Creep

**Figure 9:**
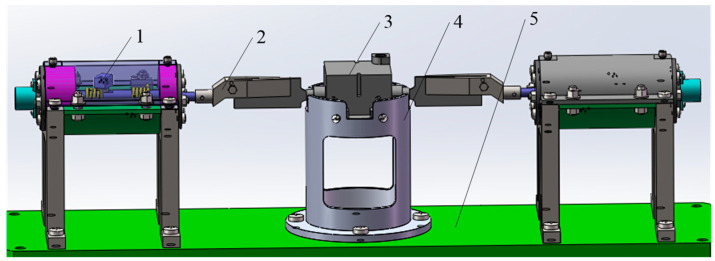
Torsion spring load table overall structure (1—unilateral loading mechanism; 2—gripper; 3—steering gear; 4—steering gear fixed seat; 5—bottom plate).

The structure of the cylindrical helical torsional spring loading table is shown in Figure 9, and is mainly composed of two identical unilateral loading mechanisms, a bottom plate, and a steering gear fixed seat. The unilateral loading mechanisms are fixed onto the bottom plate through support legs, and the rudder wing is locked by a bolt onto the gripper. The steering gear is installed on the steering gear seat and is locked by bolts. The deflection of the rudder wing is driven by the clamping claw to rotate the rotating shaft of the two unilateral loading mechanisms, and the torque is provided by the torsion springs in the unilateral loading mechanisms.

**Figure 10:**
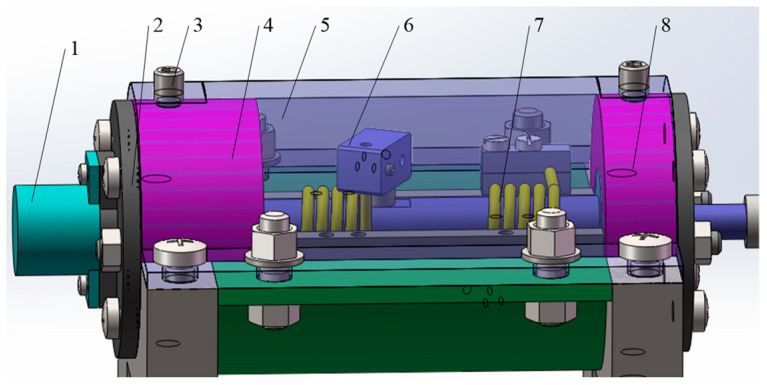
Unilateral loading structure internal structure (1—Angle sensor; 2—sensor seat; 3—fastening screws; 4—tail bearing seat; 5—stop cover; 6—torsional spring loading block; 7—torsional spring; 8—front bearing seat).

The internal structure of a unilateral loading mechanism is shown in Figure 10. The torsional spring loading platform mainly realizes the change of loading torque by replacing the unilateral loading mechanism. Different torsional springs correspond to different torque.

**Figure 11:**
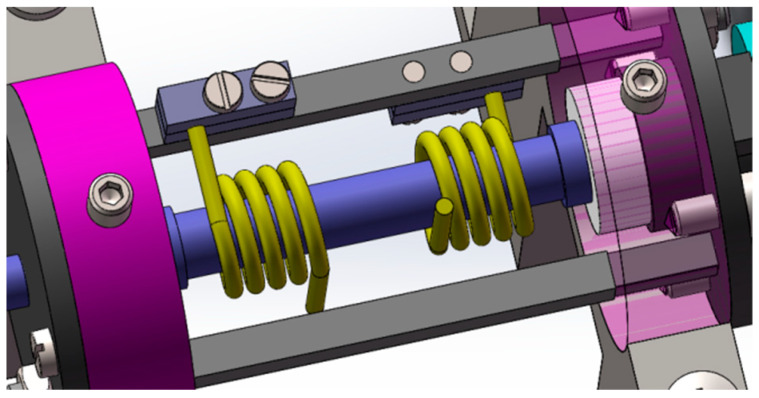
Double torsion spring installation diagram.

Each unilateral loading mechanism contains two torsion springs that are arranged in a coaxial reverse direction, and the rotating ends of the two torsion springs wind in opposite directions. The installation diagram is shown in Figure 11. Both torsion springs are pre-compressed by 30°. When the axial rotation is on one side, the force of one torsion spring increases and the compression Angle increases, while the compression Angle of the other torsion spring decreases.

The rotation of the steering gear is transmitted to the torsion springs through the clamping claw, which provides the corresponding torque. The change of the output voltage of the rotary potentiometer is measured by a multimeter, and the rotation angle can be obtained by a certain conversion formula.

**Figure 12:**
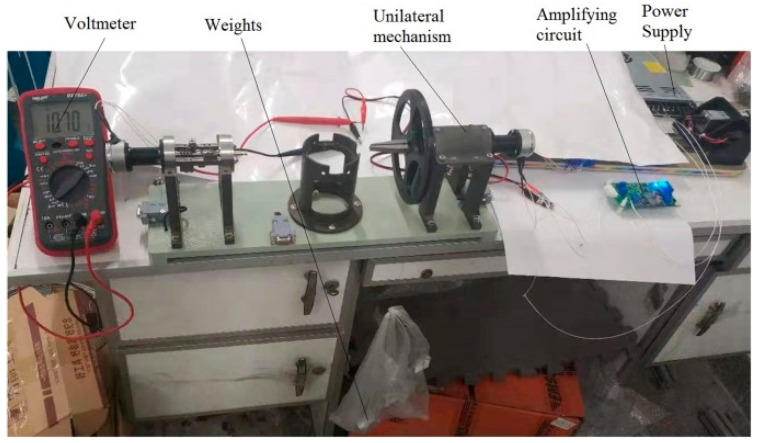
Creep test of spring on load table.

The specific experimental process is shown in Figure 12.

After loading the weight, the output voltage value of the Angle sensor can be measured by keeping the weight unchanged. After converting the voltage value into Angle, the relationship of the spring shape variable with time and the spring creep curve can be obtained.

## 3. Experimental Data and Analysis

**Figure 13:**
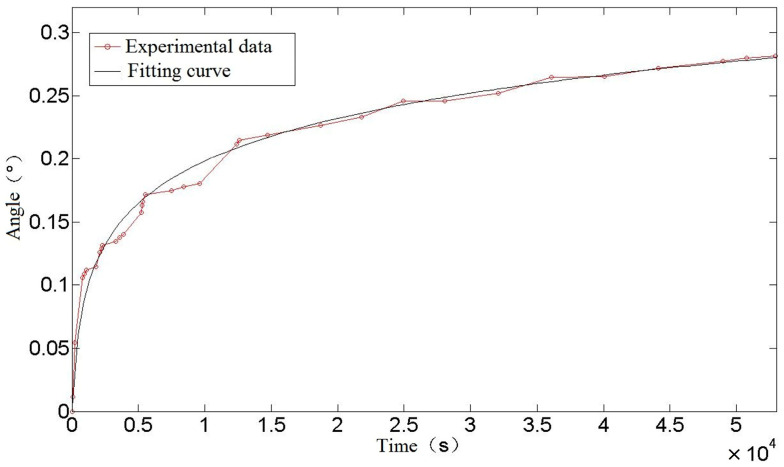
Creep curve of torsion spring.

After loading the weight, the angle change value can be obtained through the voltage change value of the voltmeter, and the experimental data are show in Table 3:

In order to facilitate direct observation, the data in the table were fitted and plotted, as shown in Figure 13.

It can be seen from Figure 13 that the creep test data and its fitting to a curve of a torsion spring conform to a typical creep curve. In the early stage of creep, the data fluctuate greatly, but in the later stage, the data tend to be stable. It is possible that, in the process of spring creep, after the sliding dislocation moves to barriers such as the grain boundary or second phase particles, the movement of dislocation stops gradually under the obstruction, and the phenomenon of accumulation appears. At this time, the number of moving dislocations decreases, which is reflected in the decrease of creep rate, or even stagnation. As more and more accretion occurs, the dislocation will climb and slide over the barrier, which may be the reason for the step phenomenon in the creep experiment.

**Figure 14:**
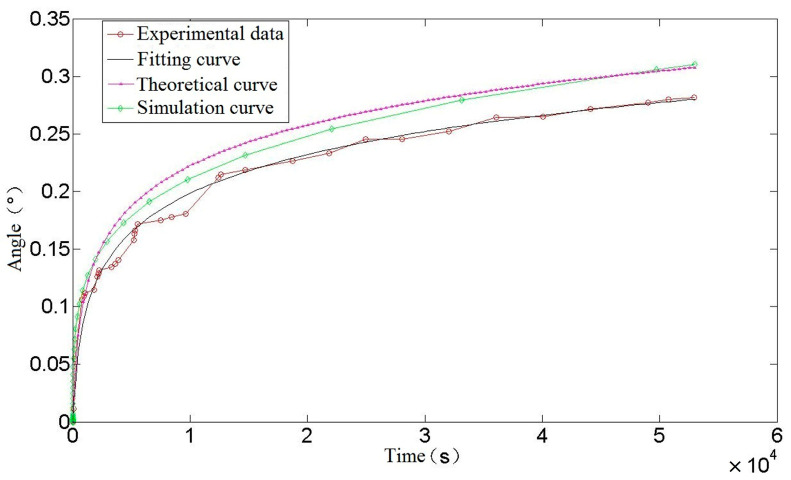
Comparison of creep angle curve of torsion spring.

Figure 8, in the previous section, shows a comparison between a theoretical and simulated curve of torsion spring creep Angle. A comparison between theory, simulation, and experimental results is shown in Figure 14.

As can be seen from Figure 14, the trend in creep strain for the theoretical calculation and finite-element simulation is the same as that of the experiment, and they all enter the stable creep stage at about 15,000 s. The theoretical calculation curve is highly consistent with the experimental fitting curve. At the initial stage of creep, the theoretical calculation curve basically coincides with the experimental data, and at the later stage of creep, the theoretical calculation creep rate is not much different from the experimental fitting curve. On the one hand, the source of error is the loss of precision caused by omitting some tiny quantities in the derivation of the theoretical calculation formula, on the other hand, it may be due to the slightly different properties of spring materials and materials in the data. At 54,000 s, the theoretical calculation is 4.3% larger than the experimental data creep value, and the error is acceptable in practical engineering application, which shows the accuracy of the theoretical calculation of torsion spring creep. As such it can be used to predict and estimate the performance and life of a spring in use, so as to avoid creep failure under high stress or long-term load conditions, thus ensuring the reliability and safety of mechanical components.

## 4. Conclusions

In this paper, the creep effect of a cylindrical helical torsion spring under pre-compression is studied. Firstly, through deconstruction and reorganization, using the original room-temperature creep constitutive equation as a basis, a room-temperature creep constitutive equation of a torsion spring can be obtained based on macroscopic tensile tests. Through the stress–strain analysis of a spring, the relationship between creep strain and time and stress of a cylindrical helical torsion spring is obtained, and the correctness of the theoretical calculation is verified by finite-element simulation.

Finally, in order to determine the influence of spring creep effect, a creep strain experiment of a torsion spring is carried out. Through experiments, it can be concluded that the cylindrical helical torsion spring enters a stable creep stage after about 15,000 s, and the creep strain angle is 0.28° after 54,000 s. By comparing the theoretical calculation results with the experimental results, it can be concluded that the error between the theoretical formula calculation results and the experimental results of a torsion spring is 4.3%, which is less than the engineering error range of 5%, which meets the requirements of engineering measurement and is of great significance for more accurately predicting and estimating the performance and life of a spring in use, and designing high-performance and reliable spring elements.

## Figures and Tables

**Table 1:** Mechanical properties of 65 Mn Steel.

Elastic modulus/(MPa)	211,000
Poisson’s ratio	0.288
Density/(t/mm^3^)	$7.83\times 10^{-9}$
$\mathrm{Tensile}\;\mathrm{strength}\;\sigma_{b}$/(MPa)	1420
$\mathrm{Yield}\;\mathrm{strength}\;\sigma_{s}$/(MPa)	1136
$\mathrm{Fatigue}\;\mathrm{limit}\;\sigma_{-1}$/(MPa)	639

**Table 2:** Main parameters of a torsion spring bearing the torque of 300 N∙mm.

Torsion spring wire diameter/(mm)	1.8
Mean diameter of coil/(mm)	11
Total number of coils	4
Free angle/(°)	120
Torsion spring force arm/(mm)	15
Torsion spring pitch/(mm)	2.5
Torsion spring helix angle/(°)	4.14

**Table 3:** Creep strain data sheet of torsion spring.

Time (s)	Angle (°)	Time (s)	Angle (°)
0	0	7500	0.174726
60	0.011457	8400	0.177591
210	0.054423	9600	0.180455
780	0.105982	12,420	0.211963
960	0.108846	12,600	0.214828
1080	0.11171	14,700	0.218987
1800	0.114575	18,700	0.226679
2100	0.126032	21,800	0.233096
2220	0.128897	24,900	0.245713
2280	0.131761	28,000	0.245677
3300	0.134625	32,100	0.2519
3600	0.13749	36,120	0.264493
3900	0.140354	40,120	0.265124
5200	0.15754	44,120	0.271456
5300	0.163269	49,000	0.277328
5340	0.166133	50,800	0.279596
5520	0.171862	54,000	0.281319

## Data Availability

Not applicable.
